# PD241 Supporting Patient Groups To Enhance Their Input To Health Technology Assessment

**DOI:** 10.1017/S0266462324004586

**Published:** 2025-01-07

**Authors:** Daniel Cairns, Kate Russell, Jackie McCormack, Jennifer Dickson

## Abstract

**Introduction:**

The Scottish Medicines Consortium (SMC) patient group submission process includes a written submission of patient experience evidence from Patient Group Partners (PGPs). To maximize the relevance and quality of information included in submissions, the first draft is reviewed by the Public Involvement Team and feedback is provided. The submission is then amended by the submitting PGP before final submission.

**Methods:**

Upon receiving a new written patient group submission, the Public Involvement Team reviews it and provides feedback highlighting any evidence gaps and areas where wording could be amended to strengthen the patient voice. The draft submission is then amended by the submitting PGP, with support from the SMC reviewer. Once finalized, the submission is collated into the body of evidence used by the SMC Committee members to assess a new medicine. The satisfaction of participating PGPs is continually assessed by an online survey. During the 2021 to 2023 period, the SMC received 232 patient group submissions, with 77 percent being amended and strengthened after receiving feedback.

**Results:**

The feedback and collaboration on amendments to draft submissions has improved the quality of patient experience evidence submitted to the SMC by PGPs. PGPs value their draft evidence being reviewed by the SMC Public Involvement Team prior to final submission. This approach resulted in high levels of trust in and satisfaction with how the SMC involves patient representatives in medicine assessments. In 2022, 89 percent of the 28 PGPs surveyed were very satisfied and 11 percent were satisfied with the support provided during the submission process.

**Conclusions:**

Evaluation of the SMC’s formal process to provide input and feedback on draft written patient group submissions demonstrates high levels of satisfaction with how the SMC works in partnership with PGPs. This approach has strengthened written evidence of patient experiences for SMC assessments. Furthermore, it helps to build and maintain a partnership approach in how the SMC works with PGPs.

